# Synthesis and characterization of a novel carboxyl group containing (co)polyimide with sulfur in the polymer backbone

**DOI:** 10.3762/bjoc.8.88

**Published:** 2012-05-25

**Authors:** Miroslav Mrsevic, David Düsselberg, Claudia Staudt

**Affiliations:** 1 Department of Organic and Macromolecular Chemistry, Heinrich-Heine University Düsseldorf, Universitätsstr.1, 40225 Düsseldorf, Germany, Fax: +492118110696

**Keywords:** DABA, functional (co)polyimides, optical polymers, sulfur-containing polymers, transparency

## Abstract

Soluble functional (co)polyimides are of great interest in the area of separation processes or optical applications, due to their excellent mechanical-, thermal- and optical properties, their superior processability and the ability to adapt their properties to a wide range of special applications. Therefore, two series of novel (co)polyimides containing fluorinated sulfur- and carboxylic acid groups consisting of 4,4′-(hexafluoroisopropylidene)di(phthalic anhydride) (6FDA), 3,5-diaminobenzoic acid (DABA), 4,4′-diaminodiphenylsulfide (4,4′-SDA) and 3,3′-diaminodiphenylsulfone (3,3′-DDS) were synthesized in a two-step polycondensation reaction. The synthesized copolymers were characterized by using NMR, FTIR, GPC, and DSC. Furthermore, with regard to processing and potential applications, the thermal stability, solubility in common organic solvents, moisture uptake, and transparency were investigated. Compared to commercially available transparent polymers, i.e., polymethylmethacrylate and cycloolefin polymers, the sulfur (co)polyimides containing carboxyl groups showed much higher glass-transition temperatures, comparably low moisture uptake and high transmission at the sodium D-line. Furthermore, good solubility in commonly used organic solvents makes them very attractive as high-performance coating materials.

## Introduction

Over the past few years, various new applications have drawn the attention of industry to materials with superior mechanical, thermal and chemical properties. High-performance polymers, such as polyimides, are renowned for comprising all these features in a remarkable way [1–2]. Additionally, their outstanding resistance to all kinds of acids makes them suitable for a broad range of applications, such as insulating technologies and industrial carriage equipment [3–4]. Furthermore, polyimides are of great interest for many membrane-based separation processes of gaseous, vaporous or liquid mixtures [5–7]. While polyimides without any functionalization already show good separation properties, they are susceptible to swelling phenomena, which lead to a significant loss in mechanical stability. As a result, the separation properties decrease sharply [8]. Swelling or sorption is not solely an important issue in separation processes, but also in optical applications in which polymeric lenses are used. The major concern in this case is moisture uptake from the air leading to a change in optical properties, which often causes undesired optical aberrations [9]. It has been shown that cross-linking can significantly inhibit swelling phenomena in membranes prepared from DABA (3,5-diaminobenzoic acid) functionalized (co)polyimides [10–12]. This approach is assumed to be appropriate for various applications. Among optical polyimides, the special class of sulfur-containing (co)polyimides is known to exhibit high refractive indices due to the high polarizability of the sulfur atoms in the polymeric backbone [13–17]. Therefore, this class is highly attractive for optical lenses and optoelectronic applications, such as OLEDs (organic light-emitting diodes) or high-performance CMOS-CISs (complementary metal–oxide–semiconductor contact image sensor) [18]. Although (co)polyimides exhibit excellent properties and are numbered among the high-performance polymer materials, many polyimides are not soluble in common organic solvents, which makes their direct processing and large-scale manufacturing difficult. In this case, processing of the polyamic acid followed by heat treatment for imidization is necessary. In contrast, polyimides containing fluorinated monomers, such as 6FDA (4,4′-(hexafluoroisopropylidene)di(phthalic anhydride)) are known to exhibit excellent solubilities in common organic solvents [19]. Because of the aforementioned properties of sulfur-containing polyimides and fluorinated polyimides, a (co)polyimide with a high refractive index and simultaneously good solubility in organic solvents appears to be very valuable. If this material additionally acquires functional groups, i.e., carboxyl groups of the 3,5-diaminobenzoic acid (DABA) monomer, cross-linking is possible in order to decrease the water sorption. It is also possible to use carboxyl groups to attach inorganic, high-refractive-index materials, such as ITO (indium tin oxide) or TiO_2_, to the polymer backbone to further improve the refractive index of such a (co)polyimide [20–22].

In this work, we report on the synthesis and characterization of fluorinated, DABA- and sulfur-containing (co)polyimides. These (co)polyimides, which were synthesized from a combination of different monomers with special properties, show excellent solubilities, very low water sorptions, and very good optical properties, making them highly attractive for optical applications.

## Results and Discussion

### Preparation of (co)polyimides

**Scheme 1:**
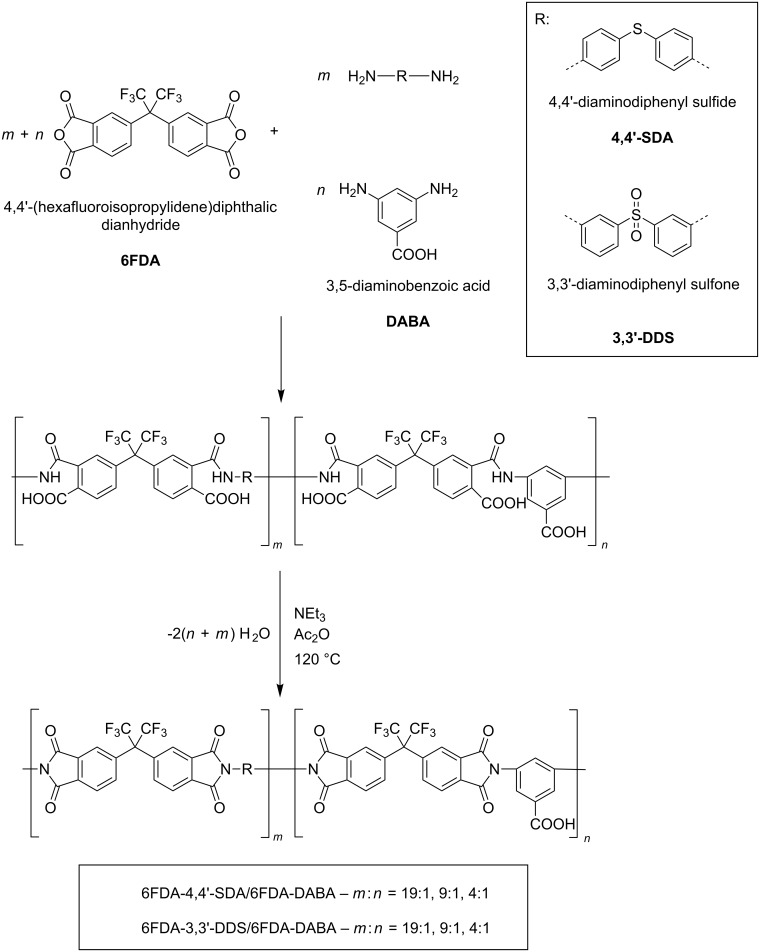
General synthesis scheme for the (co)polyimides investigated in this work.

The syntheses of the polyimides and (co)polyimides were performed in a two-step polycondensation reaction as shown in Scheme 1, analogous to that of Sroog et al. [23] and that previously described by our group [17]. In this work, chemical imidization was used exclusively, because previous work of our group showed that the imidization degree of chemically converted PAAs is superior to thermally imidized PAAs [24]. Aromatic diamines as shown in Scheme 1, carrying either sulfone (3,3′-diaminodiphenyl sulfone, 3,3′-DDS), or (as partly described in a previous work from our group) sulfide (4,4′-diaminodiphenyl sulfide, 4,4′-SDA) groups, were used for the synthesis of novel (co)polyimides in this work [25]. In order to investigate structural effects of the DABA groups on the glass-transition temperature, molecular weight, transparency and water sorption, both polymers 6FDA-4,4′-SDA/6FDA-DABA *m*:*n* and 6FDA-3,3′-DDS/6FDA-DABA *m*:*n* were synthesized with three different diamine ratios, *m*:*n* = 19:1, 9:1 and 4:1.

### ^1^H NMR spectroscopy

**Figure 1:**
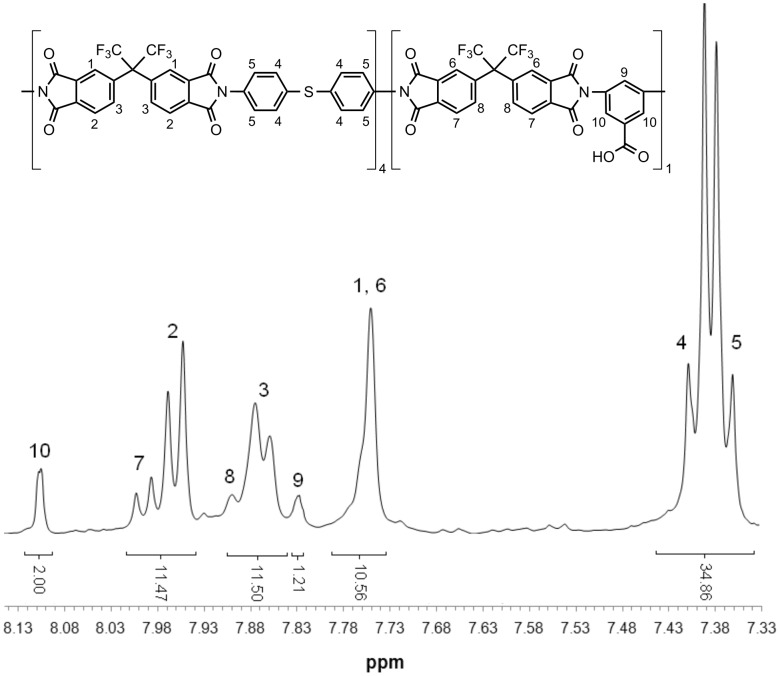
Aromatic section of the ^1^H NMR spectra of 6FDA-4,4′-SDA/6FDA-DABA, *m*:*n* = 4:1.

**Figure 2:**
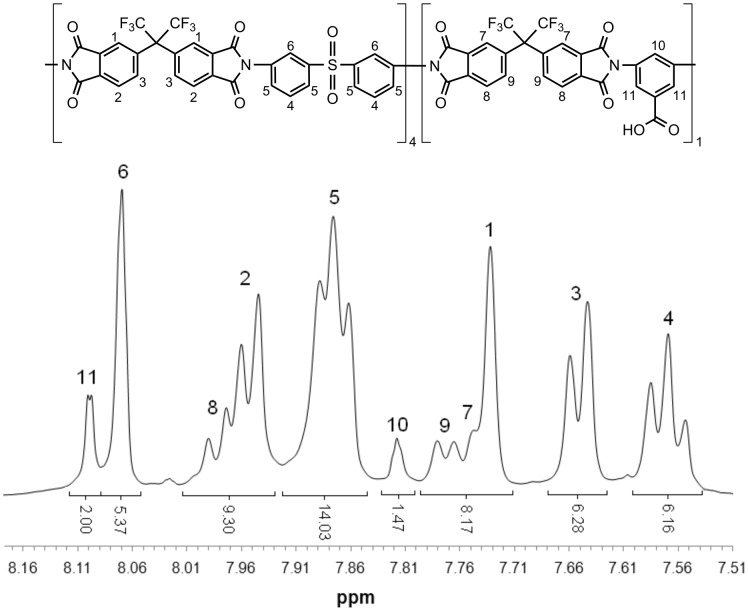
Aromatic section of the ^1^H NMR spectra of 6FDA-3,3′-DDS/6FDA-DABA, *m*:*n* = 4:1.

The compositions of the polymers synthesized were proven by ^1^H NMR spectral analysis. The integrals of the protons belonging to the diamines are in good accordance with the desired composition of the structure. Since all protons of both polymers are exclusively aromatic, only the aromatic section of the spectra is of interest and is exemplarily shown in Figure 1 and Figure 2 for the (co)polyimides 6FDA-4,4′-SDA/6FDA-DABA, *m*:*n* = 4:1, and 6FDA-3,3′-DDS/6FDA-DABA, *m*:*n* = 4:1. For all other (co)polyimides that have been synthesized in this work, similar agreement in the expected areas was found.

**Figure 3:**
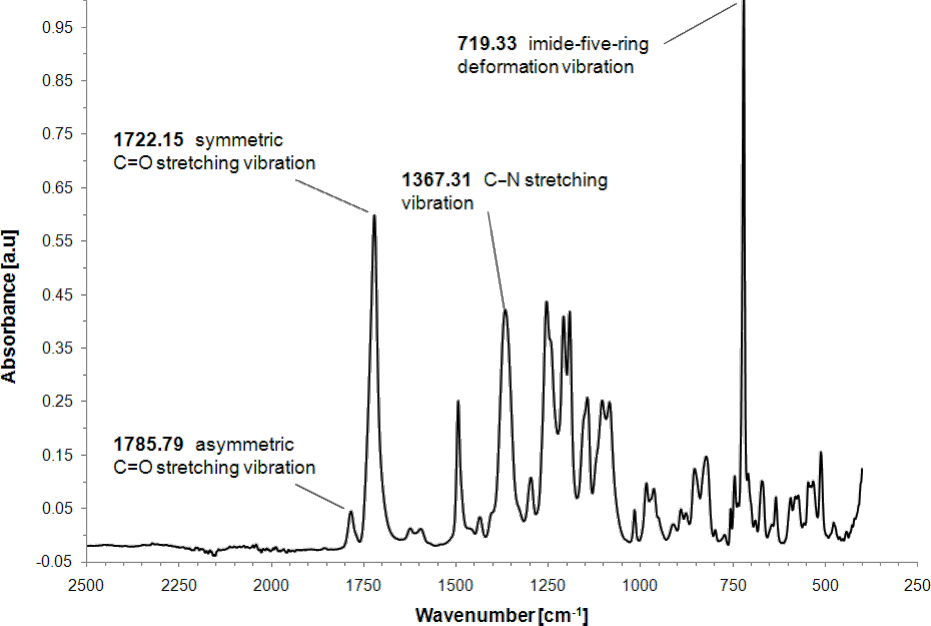
FTIR spectrum of 6FDA-4,4′-SDA/6FDA-DABA 4:1.

Figure 1 and Figure 2 show that it is possible to determine the real diamine ratio of the synthesized (co)polyimides by integrating and comparing the eight aromatic protons of 4,4-SDA (Figure 2; *H*4, *H*5) or 3,3′-DDS (Figure 3; *H*4, *H*5, *H*6) with the two ortho-substituted DABA-protons (Figure 2; *H*10 and Figure 3; *H*11). Table 1 lists the expected diamine ratios of the synthesized (co)polyimides and those determined by ^1^H NMR spectroscopy. Table 1 shows that the diamine ratios determined by ^1^H NMR spectroscopy are in good agreement with the expected diamine ratios, with relative deviations of 15%.

**Table 1:** Expected diamine ratios of the (co)polyimides and diamine ratios determined by ^1^H NMR spectroscopy.

(co)polyimide composition and expected diamine ratio		diamine ratio measured by ^1^H NMR

6FDA-4,4′-SDA/6FDA-DABA	19:1	21:1
6FDA-4,4′-SDA/6FDA-DABA	9:1	10:1
6FDA-4,4′-SDA/6FDA-DABA	4:1	4:1
6FDA-3,3′-DDS/6FDA-DABA	19:1	16:1
6FDA-3,3′-DDS/6FDA-DABA	9:1	8:1
6FDA-3,3′-DDS/6FDA-DABA	4:1	4:1

### FTIR measurements

**Figure 4:**
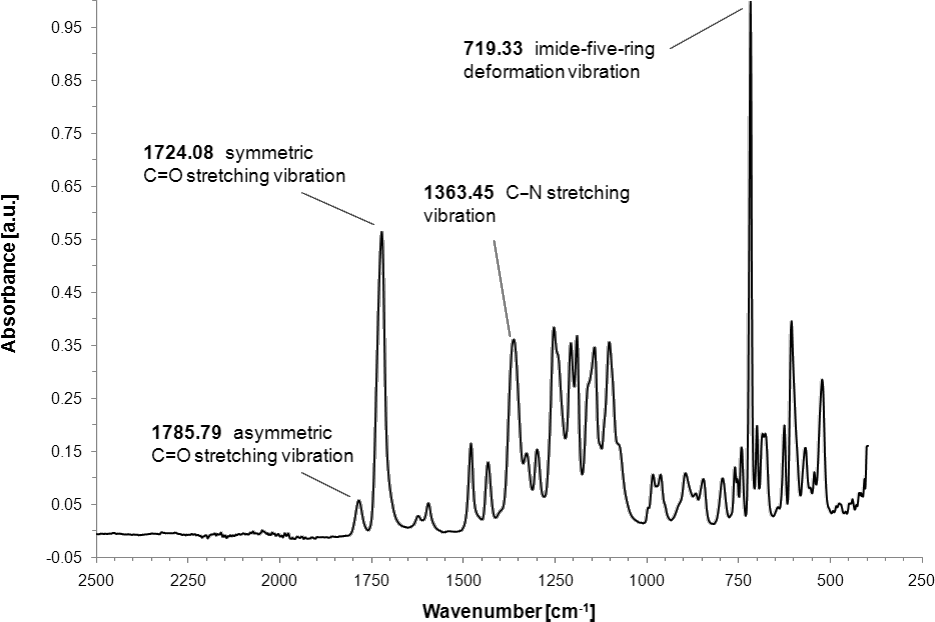
FTIR spectrum of 6FDA-3,3′-DDS/6FDA-DABA 4:1.

The structures of the synthesized (co)polyimides were further verified by FTIR spectroscopy. In Figure 3 and Figure 4, the FTIR spectra of 6FDA-4,4′-SDA/6FDA-DABA 4:1 and 6FDA-3,3′-DDS/6FDA-DABA 4:1, respectively, are shown. Polyimides in general, exhibit characteristic vibration bands, i.e., the asymmetric C=O stretching vibration, the symmetric C=O stretching vibration, the C–N stretching vibration, and the imide-five-ring deformation vibration. As shown in Figure 3, the characteristic bands of 6FDA-4,4′-SDA/6FDA-DABA 4:1 are, as expected, located at 1785.79 cm^−1^ (asymmetric C=O stretching vibration), 1722.15 cm^−1^ (symmetric C=O stretching vibration), 1367.31 cm^−1^ (C–N stretching vibration) and 719.33 cm^−1^ (imide-five-ring deformation vibration). Similar results were found for the sulfone-containing 6FDA-3,3′-DDS/6FDA-DABA 4:1, which is shown in Figure 4. All synthesized (co)polyimides showed the expected vibrational bands.

### GPC measurements

Gel permeation chromatography (GPC) measurements of the synthesized (co)polyimides were performed in order to determine the average of the molecular weight (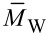). Table 2 shows the 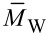 of the synthesized (co)polyimides determined by UV detection.

**Table 2:** Results of the GPC measurements for the synthesized (co)polyimides.

(co)polyimide		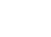 [g·mol^−1^]

6FDA-4.4'-SDA/6FDA-DABA	19:1	77,000
6FDA-4.4'-SDA/6FDA-DABA	9:1	35,000
6FDA-4.4'-SDA/6FDA-DABA	4:1	26,000

6FDA-3,3′-DDS/6FDA-DABA	19:1	30,000
6FDA-3,3′-DDS/6FDA-DABA	9:1	36,000
6FDA-3,3′-DDS/6FDA-DABA	4:1	34,000

The synthesized (co)polyimides showed average molecular weights between 26,000 and 77,000 g·mol^−1^. The molecular weights of the sulfide-containing (co)polyimides showed a significant decrease with increasing DABA content. This correlation is probably due to a strong −I effect of the carboxylic group of the DABA monomer used in the polymerization reaction, which deactivates the amino groups and inhibits their nucleophilic character, leading to a slower buildup of 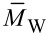 during the polyaddition reaction.

### DSC measurements

Differential scanning calorimetry (DSC) is a method to characterize the thermal properties of polymers and is also useful in disclosing composition based correlations. In order to do so, the glass-transition temperatures of all (co)polyimides synthesized were determined (Table 3). Additionally, glass-transition temperatures of the homopolymers 6FDA-4,4′-SDA and 6FDA-3,3′-DDS, previously investigated in our group [24], and of 6FDA-DABA [26] for which data were taken from the literature, were listed to verify the correlations. Additionally, the *T*_g_ values of the aforementioned polymers were compared to those of the 4MPD (2,3,5,6-tetramethyl-1,4-phenylenediamine) containing 6FDA-4MPD/6FDA-DABA *m*:*n* (co)polyimide, which has already been investigated in several membrane-based separation processes [7,27–29].

**Table 3:** Glass-transition temperatures *T*_g_ of the synthesized (co)polyimides and their respective homopolymers.

(co)polyimide	*T*_g_ [°]

6FDA-4.4'-SDA [24]	301
6FDA-4.4'-SDA/6FDA-DABA 19:1	301
6FDA-4.4'-SDA/6FDA-DABA 9:1	305
6FDA-4.4'-SDA/6FDA-DABA 4:1	311
6FDA-DABA [26]	348

6FDA-3.3'-DDS [24]	270
6FDA-3.3'-DDS/6FDA-DABA 19:1	273
6FDA-3.3'-DDS/6FDA-DABA 9:1	276
6FDA-3.3'-DDS/6FDA-DABA 4:1	282
6FDA-DABA [26]	348

6FDA-4MPD [27]	425
6FDA-4MPD/6FDA-DABA [28] 19:1	424
6FDA-4MPD/6FDA-DABA [29] 9:1	419
6FDA-4MPD/6FDA-DABA [7] 4:1	411
6FDA-DABA [26]	348

*T*_g_ values far above 270 °C were found for all synthesized (co)polyimides, as expected. Gibbs et al. already showed in 1958, that the glass-transition temperature is affected by the rigidity of the polymer backbone [30] and that the glass-transition temperature of a copolymer is a linear function of the composition of the copolymer [31]. Thereby, certain molecular units, such as freely rotatable single bonds, are able to decrease the rigidity of the polymer chain, while double bonds or sterically demanding groups increase the stiffness of the polymer chain. Furthermore, Endrey showed that the position of aromatic substituents has a significant influence on the polymer rigidity [32]. It was disclosed that polyimides prepared from para-substituted aromatic diamines exhibit higher stiffness than those made from meta-substituted aromatic diamines.

**Figure 5:**
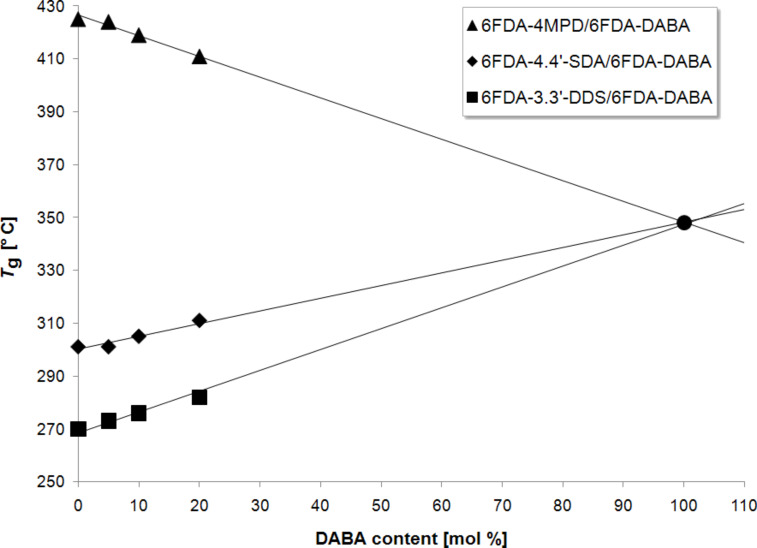
Glass-transition temperatures of the synthesized (co)polyimides, their respective homopolymers [24,26–27] and a series of reference (co)polyimides [7,28–29].

The results shown in Table 3 and Figure 5 are perfectly in line with the expected, above-mentioned behavior. Apparently, the sulfide-containing (co)polyimides exhibit higher *T*_g_ values than the sulfone-containing (co)polyimides. This is due to the differently substituted aromatic diamines. The para-substituted 4,4′-SDA induces a higher stiffness compared to the meta-substituted 3,3′-DDS, which results in higher *T*_g_ values for this copolymer series. Additionally, Figure 5 shows that the *T*_g_ values of both synthesized (co)polyimide series increase with increasing DABA content and follow the predicted linear trend. The reason for this increase is that the DABA monomer exhibits fewer freely rotatable single bonds than both of the sulfur-containing diamines, and this therefore improves the rigidity of the polymer chain. Additionally the carboxyl groups of the DABA can lead to hydrogen bonding in the polymer chains, which additionally increases the glass-transition temperature.

For further verification of the postulated effects, the results obtained for the synthesized (co)polyimides were compared to those of the reference copolymer 6FDA-4MPD/6FDA-DABA *m*:*n*. The *T*_g_ values of this (co)polyimide correlate very well with the postulations from Gibbs et al. and Endrey [30–32]. The glass-transition temperatures decrease linearly with increasing DABA content, which can be explained by two corresponding effects, both of which improve the stiffness of the polymer chain. The first one is the para-substitution of the 4MPD monomer in contrast to the meta-substitution of the DABA monomer, and the second one is the sterically demanding methyl groups of the 4MPD monomer, which hamper the rotatability of the single bonds.

### Solubility

The solubility of the synthesized (co)polyimides was tested qualitatively in common organic solvents. The results are listed in Table 4. All (co)polyimides exhibited high solubility in amidic solvents, such as dimethylacetamide (DMAC), dimethylformamide (DMF), and *N*-methyl-2-pyrrolidone (NMP), as well as in other non-amidic solvents, such as tetrahydrofuran (THF), dimethyl sulfoxide (DMSO) and acetone. No differences in solubility either between the different (co)polyimides or within each (co)polyimide series were observed. The high solubility of the (co)polyimides presented in this work seemed to be a result of the –CF_3_ groups of the 6FDA, which was used as a dianhydride. Such highly soluble polymers are invaluable for the chemical industry when it comes to processing. Especially, spin- or dip-coating can be easily realized with such polymers.

**Table 4:** Solubility of the (co)polyimides synthesized.^a^

solvent	6FDA-4,4′-SDA/6FDA-DABA *m*:*n*			6FDA-3,3′-DDS/6FDA-DABA *m*:*n*		
	4:1	9:1	19:1	4:1	9:1	19:1

dimethylacetamide	○	○	○	○	○	○
dimethylformamide	○	○	○	○	○	○
*N-*methyl-2-pyrrolidone	○	○	○	○	○	○
dimethyl sulfoxide	○	○	○	○	○	○
tetrahydrofurane	○	○	○	○	○	○
acetone	○	○	○	○	○	○
toluene	×	×	×	×	×	×
cyclohexane	×	×	×	×	×	×
dichloromethane	×	×	×	×	×	×
*n*-hexane	×	×	×	×	×	×

^a^The solubility was determined at 10% solid content. ○: soluble; the solid polymer was completely dissolved in the solvent. ×: insoluble; dissolution of the polymer was <1%.

### Moisture uptake

As mentioned, water sorption caused by atmospheric moisture uptake can cause a severe problem in optical applications, since this leads to changes of the refractive index, and as a result undesired optical aberrations are observed. In order to characterize the moisture uptake of the synthesized (co)polyimides, measurements in a climate cabinet on (co)polyimide films were performed. To prepare these (co)polyimide film samples, the following procedure was applied:

#### Sample preparation for moisture-uptake measurements

Dry (co)polyimide powder (450 mg) was dissolved in 10 mL of THF and stirred with a magnetic stirrer for 1 h. The (co)polyimide solution was filtered through a glass frit (pore size 3 μm), cast onto a metal mold with a diameter of 6 cm, and covered with a plastic funnel capped with a tissue to prevent deposition of dust particles. The solvents were evaporated at room temperature for 24 h. Free-standing film samples with an average thickness of about 150 μm were obtained. These films were detached from the metal surface by using distilled water. Afterwards, the film samples were transferred to the vacuum oven and dried at 150 °C and 80 mbar for 2–3 days.

**Figure 6:**
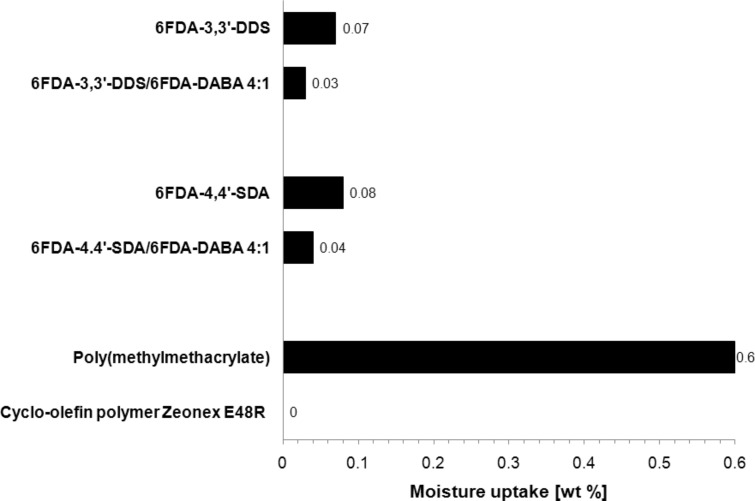
Comparison of the relative moisture uptake after one day between 6FDA-3,3′-DDS/6FDA-DABA 4:1 and 6FDA-4,4′-SDA/6FDA-DABA 4:1, their respective homopolyimides [24], and commercially available polymers [9].

The prepared film samples of the synthesized (co)polyimides and their respective homopolymers were first weighted and then exposed to 98% relative humidity at 50 °C in a climate cabinet for nine days. Once per day, the samples were weighted to determine the relative moisture uptake. Maximum moisture uptake for all polyimides and (co)polyimides was found after the first day, which may be due to surface adsorption processes. Afterwards, no further moisture uptake was found. In Figure 6, the moisture uptake results of the 6FDA-3,3′-DDS/6FDA-DABA 4:1 and 6FDA-4,4′-SDA/6FDA-DABA 4:1 are exemplarily compared to their respective homopolymers and also to commercially available optical polymers, such as poly(methylmethacrylate) and the recently developed cyclo-olefin polymer Zeonex E48R^®^, which were measured under similar conditions (50 °C, 90% relative humidity) [9].

As shown in Figure 6, the synthesized DABA-containing (co)polyimides exhibited lower moisture uptakes than their respective homopolyimides. This is a surprising result, since it was assumed that the integration of carboxylic acid groups in the polymer backbone would increase the moisture uptake due to the increased hydrophilic properties compared to the homopolymers. The reason for this behavior may be the formation of hydrogen bonds between the polymer chains, which leads to sorption-inhibiting polymer networks. The relative decrease of the moisture uptake of the DABA-containing (co)polyimides compared to their respective homopolymers is 43% in the case of 6FDA-3,3′-DDS/6FDA-DABA 4:1 and 50% in the case of 6FDA-4,4′-SDA/6FDA-DABA 4:1. Compared to commercially available polymers, it can be stated that the moisture uptake of both (co)polyimides is lower by at least one order of magnitude compared to that of poly(methylmethacrylate). The cyclo-olefin polymer Zeonex E48R^®^, however, shows the lowest moisture uptake, but unfortunately has disadvantages in terms of thermal stability, which makes it less attractive for high-temperature applications. Table 5 shows the relative moisture uptake of both synthesized *m*:*n* = 4:1 (co)polyimides and the commercially available polymers with regard to their glass-transition temperature. It is obvious that DABA-containing (co)polyimides exhibit much higher glass-transition temperatures and very low moisture uptake, which makes them attractive as components for high-temperature applications, such as light emitting diodes (LEDs) [34].

**Table 5:** Relative moisture uptake and glass-transition temperatures of synthesized (co)polyimides and commercial polymers used in optical applications.

polymer	moisture uptake	*T*_g_
	[wt %]	[°C]

6FDA-4,4′-SDA/6FDA-DABA 4:1	0.04	311
6FDA-3,3′-DDS/6FDA-DABA 4:1	0.03	282
PMMA	0.60 [9]	106 [33]
Zeonex E48R^®^	0.00 [9]	139 [33]

### UV–vis spectroscopy

**Figure 7:**
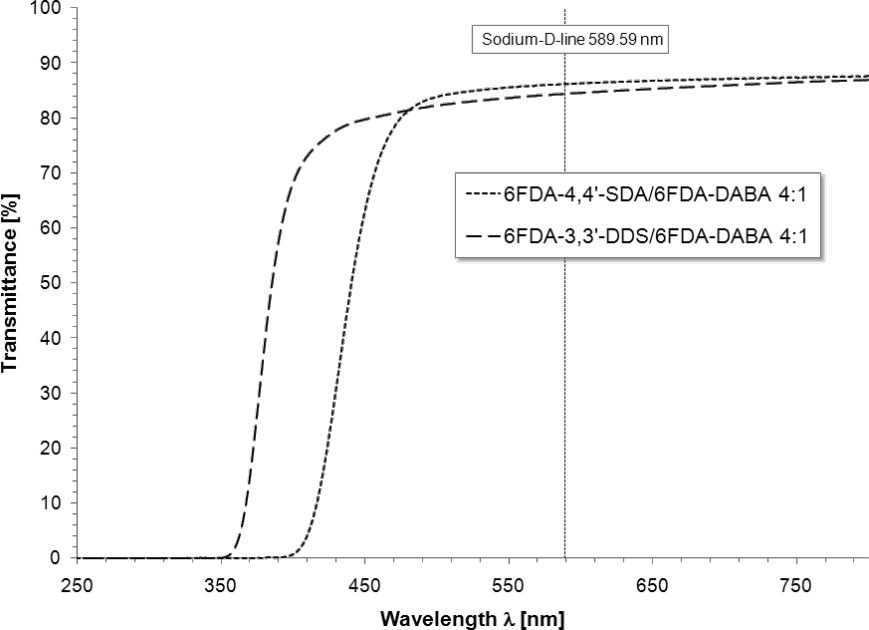
Transmittance UV–vis spectra of 6FDA-4,4′-SDA/6FDA-DABA 4:1 and 6FDA-3,3′-DDS/6FDA-DABA 4:1.

In order to characterize the optical properties of the (co)polyimides with respect to their color and transparency, UV–vis spectra of free standing films were recorded. The previously described procedure to prepare free-standing films for moisture uptake measurements was also applied for UV–vis spectroscopy. Spectra were recorded in the range of 250 to 800 nm with average film thicknesses of 140 μm. To ensure sufficient comparability, transparency is defined as the transmittance value at the wavelength of the Fraunhofer sodium D-line (589.59 nm) [9]. Using this method, very good transparency with transmittance over 86% for 6FDA-4,4′-SDA/6FDA-DABA *m*:*n* (co)polyimides and 84% for 6FDA-3,3′-DDS/6FDA-DABA *m*:*n* (co)polyimides was observed. Figure 7 shows the transmittance UV–vis spectra of 6FDA-4,4′-SDA/6FDA-DABA 4:1 and 6FDA-3,3′-DDS/6FDA-DABA 4:1 representatively for all synthesized polymers.

In order to characterize a material regarding its color, the cutoff wavelength is a useful tool. It is defined as the wavelength at 50% transmittance. If this value is higher than 400 nm, the material is considered to be colored. As shown in Figure 7, the 6FDA-3,3′-DDS/6FDA-DABA 4:1 (co)polyimides exhibit cutoff wavelengths below 400 nm and are therefore perfectly colorless. Similar cutoff wavelengths have also been found for the 19:1 and 9:1 (co)polyimides. In contrast, the 6FDA-4,4′-SDA/6FDA-DABA *m*:*n* (co)polyimides show cutoff wavelengths over 400 nm and were yellow in color. Compared to their respective homopolymers 6FDA-3,3′-DDS (84%) and 6FDA-4,4′-SDA (86%) [21] the (co)polyimides exhibited approximately the same transparency and color. In comparison to commercially available polymers such as polysulfone (84%) [30], similar (6FDA-3,3′-DDS/6FDA-DABA: 84%) or slightly improved (6FDA-4,4′-SDA/6FDA-DABA: 86%) transparency values were found. However, poly(methylmethacrylate) (92.5%) and Zeonex E48R^®^ (92%) exhibit higher transparency values [30] compared to the synthesized (co)polyimides.

**Figure 8:**
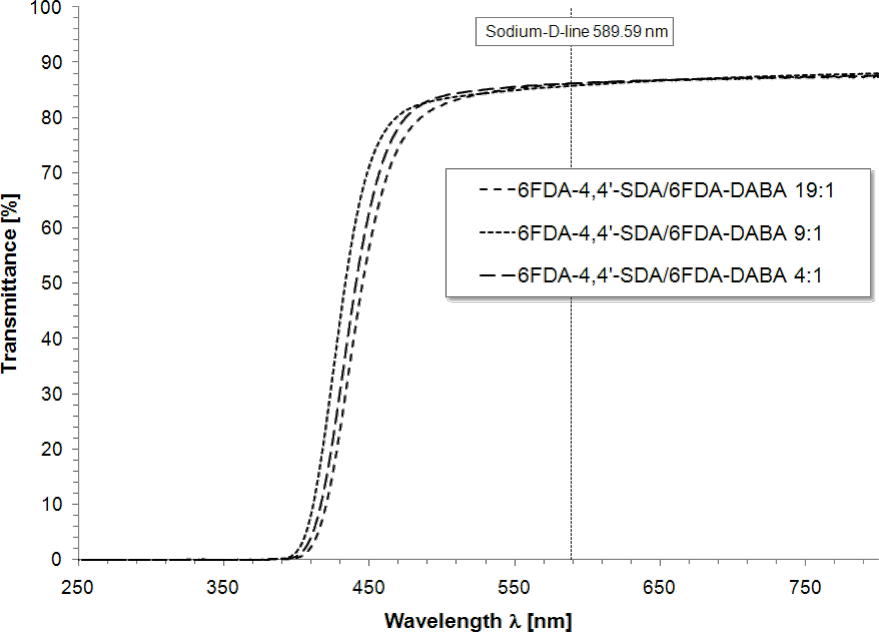
Transmittance UV**–**vis spectra of 6FDA-4,4′-SDA/6FDA-DABA (co)polyimides.

In order to verify if there is any correlation between the DABA content and the color of the (co)polyimide, the slightly yellow 6FDA-4,4′-SDA/6FDA-DABA *m*:*n* (co)polyimides with ratios of 4:1, 9:1 and 19:1 were investigated concerning their cutoff wavelengths. The results are shown in Figure 8. Figure 8 shows that the cutoff wavelengths of the (co)polyimides with different compositions were found within the error range of the measurement. No differences in transparency were observed and also no correlation was observed concerning the DABA content. The cutoff wavelengths for the different (co)polyimides were determined at 446 nm for the 19:1 (co)polyimide, 434 nm for the 9:1 (co)polyimide, and 441 nm for the 4:1 (co)polyimide.

## Conclusion

Two series of novel sulfur- and carboxylic-group-containing (co)polyimides were successfully synthesized and thoroughly characterized. All (co)polyimides were prepared by polycondensation reactions from a commercially available fluorinated dianhydride 6FDA, two sulfur-containing diamines (4,4′-SDA and 3,3′-DDS) and one diamine with a carboxylic acid (DABA). The structures of all polymers were verified by ^1^H NMR spectroscopy, FTIR spectroscopy and GPC measurements, with molecular weight averages between 26,000 and 77,000 g/mol. The (co)polyimides showed very good solubilities in common organic solvents and excellent thermal stabilities, with glass-transition temperatures between 270 °C and 311 °C, making them highly attractive for high-temperature applications. Additionally, all (co)polyimides showed very low moisture uptake, at less than 0.04 wt %, and high transparencies between 84% and 86%, which are crucial requirements for optical polymers.

## Experimental

### Materials

*N*,*N*-dimethylacetamide (DMAC), was obtained from Merck (>99%, for synthesis). The solvent was heated under reflux for a minimum of eight hours over calcium hydride for desiccation before distillation. 4,4′-diaminodiphenyl sulfide (4,4′-SDA, Sigma Aldrich) and 3,3′-diaminodiphenyl sulfone (3,3′-DDS, Sigma Aldrich) were purified by sublimation at 0.1 mbar and 190 °C. 4,4′-(hexafluoroisopropylidene)diphthalic anhydride (6FDA, Alfa Aesar) was purified by sublimation at 0.1 mbar and 215 °C. 3,5-Diaminobenzoic acid (DABA, Merck) was purified by sublimation at 0.1 mbar and 195 °C. Acetic anhydride (J.T. Baker, 97%) and triethylamine (Acros organics, 99%) were used as received.

#### General synthesis of (co)polyimides

A 100 mL Schlenk flask provided with a magnetic stir bar was set under a nitrogen atmosphere and heated up to eliminate water residues before the polymerization. The complete reaction up to the precipitation of the polyimide was performed under a nitrogen atmosphere. A mixture of diamines (4 mmol) of the desired composition was placed into the flask and dissolved with 6 mL of dried DMAC. When the diamines were dissolved completely, 1.777 g (4 mmol) of the dianhydride 6FDA and further 6 mL of DMAC were added to the solution. The solution was stirred for 4 h at room temperature. After that, 10 mL of DMAC were added to ensure continuous stirring of the viscous solution for another 24 h. For the imidization, a solution of 1.225 g (12 mmol, triple excess based on 6FDA) acetic anhydride and 1.214 g (12 mmol, triple excess based on 6FDA) triethylamine dissolved in 5 mL DMAC were added to start the chemical imidization of the polyamic acid, which was performed by heating under reflux at 120 °C (oil bath temperature) for 30 min. For this step the Schlenk flask was fitted with a reflux condenser, and the bubble counter was put on top of it. After imidization, the polyimide solution was precipitated in a stirred mixture of 250 mL ethanol and 250 mL distilled water. The received polyimide filaments were filtered, washed with ethanol, and then ground. The obtained powder was washed again with ethanol three times and air dried for 24 h. The final drying step was performed in a vacuum oven for a minimum of one week at 150 °C and 80 mbar. By using this method, six different (co)polyimides from 6FDA, the diamines 4,4′-SDA and DABA and from 6FDA, and the diamines 3,3′-DDS and DABA with diamine ratios of 19:1, 9:1 and 4:1 were synthesized.

#### Characterization

^1^H NMR spectra were recorded with a Bruker Avance DRX 500 spectrometer with Si(CH_3_)_4_ as the internal standard at room temperature in deuterated tetrahydrofuran (THF-*d*_8_). FTIR spectra were recorded at 4 cm^−1^ resolution with 1024 sample scans on a Nicolet 6700 equipped with an ATR unit. DSC measurements were performed on a Mettler Toledo DSC822 under nitrogen at a heating rate of 20 °C/min to a maximum of 400 °C. GPC measurements with tetrahydrofuran (THF) as solvent were recorded on a Viscotek VE 2001 GPC at 60 °C and a flow rate of 1.0 mL/min. UV–vis spectrometry data was recorded on a Perkin Elmer Lambda2S with air as the calibrating reference. Water sorption experiments were carried out with a Weiss Umwelttechnik WK1-180/80 climate cabinet. Experiments were carried out by measuring at 50 °C and 98% relative humidity.
